# Construction of pillar[4]arene[1]quinone–1,10-dibromodecane pseudorotaxanes in solution and in the solid state

**DOI:** 10.3762/bjoc.16.245

**Published:** 2020-12-02

**Authors:** Xinru Sheng, Errui Li, Feihe Huang

**Affiliations:** 1 State Key Laboratory of Chemical Engineering, Center for Chemistry of High-Performance & Novel Materials, Department of Chemistry, Zhejiang University, Hangzhou 310027, P. R. China; 2 Green Catalysis Center and College of Chemistry, Zhengzhou University, Zhengzhou 450001, P. R. China

**Keywords:** host–guest chemistry, pillar[4]arene[1]quinones, pillararenes, pseudorotaxanes, supramolecular chemistry

## Abstract

We report novel pseudorotaxanes based on the complexation between pillar[4]arene[1]quinone and 1,10-dibromodecane. The complexation is found to have a 1:1 host–guest complexation stoichiometry in chloroform but a 2:1 host–guest complexation stoichiometry in the solid state. From single crystal X-ray diffraction, the linear guest molecules thread into cyclic pillar[4]arene[1]quinone host molecules in the solid state, stabilized by CH∙∙∙π interactions and hydrogen bonds. The bromine atoms at the periphery of the guest molecule provide convenience for the further capping of the pseudorotaxanes to construct rotaxanes.

## Introduction

Relying on the research of basic science, supramolecular chemistry has become an important mean for constructing functional materials from the bottom up as well as an important way to create new substances with functions [1–5]. Through ingenious designs and the applications of molecular recognition and self-assembly strategies, many exquisite supramolecular architectures have been fabricated, including molecular switches, molecular logic gates, molecular machines, supramolecular polymers, etc. [6–11]. Pseudorotaxanes not only are used as the supramolecular precursors for the synthesis of rotaxanes and catenanes but also play an important role in the construction of supramolecular architectures and chemical topology [12–23]. Seeking new systems to produce pseudorotaxanes is currently considered a “hot topic” in supramolecular chemistry.

As a new class of supramolecular macrocyclic hosts, pillararenes have received extensive attention in recent years due to their unique pillar structures and rich environmental responsiveness [24–29]. There are more and more reports on pillararenes complexing with different guest molecules to construct pseudorotaxanes [30–35]. Previously, our group first demonstrated that alkyl chains can be encapsulated in the pillar[5]arene cavity, forming [2]pseudorotaxanes driven by CH∙∙∙π interactions [36–37]. This discovery facilitated the preparation of threaded structures based on the pillar[5]arene–alkyl chain recognition motif. Benefiting from these results, various compounds with different substituents on the alkyl chains have been used to prepare pseudorotaxanes with pillar[5]arenes and have opened potential applications in different fields [38–43].

**Scheme 1:**
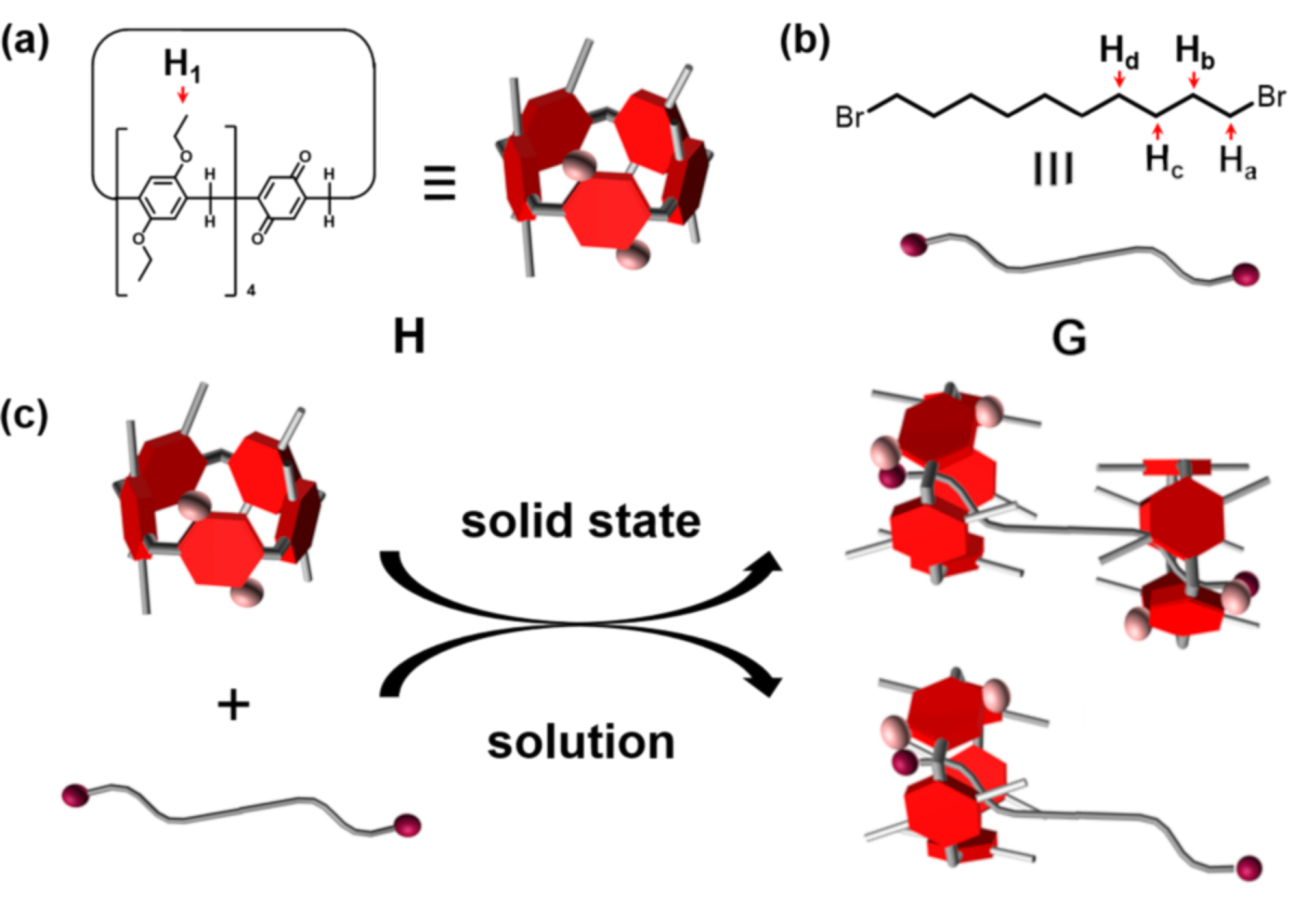
Chemical structures and schematic representation of (a) the pillar[4]arene[1]quinone **H**; (b) 1,10-dibromodecane (**G**); and (c) schematic representation of the pseudorotaxanes based on **H** and **G**.

So far, fabricating pseudorotaxanes containing more than two components is still a difficult task. Herein, we report new pseudorotaxanes based on the pillar[4]arene[1]quinone **H** and 1,10-dibromodecane (**G**, Scheme 1). The pillar[4]arene[1]quinone **H**, which is composed of four 1,4-diethoxybenzene subunits and one benzoquinone subunit, was prepared by partial oxidation of perethylated pillar[5]arene according to previous reports [44–45]. We found that **H** and **G** can be used to build a [3]pseudorotaxane in the solid state but a [2]pseudorotaxane in solution.

## Results and Discussion

### Host–guest complexation in the solid state

**Figure 1:**
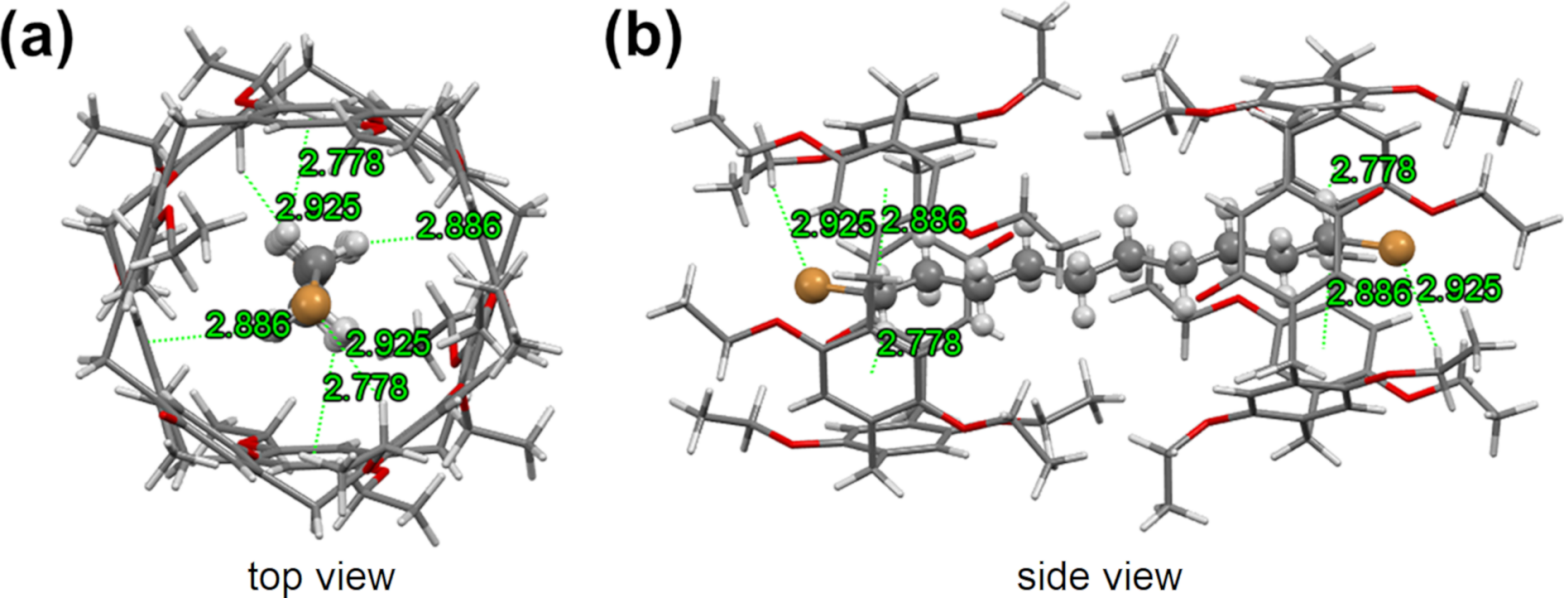
Crystal structures of the [3]pseudorotaxane composed of **H** and **G** in the solid state. Color code: C, gray; Br, orange; O, red; and H, white. CH∙∙∙π distances (Å): 2.778, 2.886, 2.778, and 2.886; CH···Br distances (Å): 2.925 and 2.925.

Cocrystals of **H** and **G** were obtained by slow evaporation of the solution in methanol. The X-ray crystallography revealed that two host molecules complex one guest molecule, forming a [3]pseudorotaxane in the solid state (Figure 1). In the crystal structure, the alkyl chain of the guest is threaded through the cavities of two host molecules, which is stabilized by multiple CH∙∙∙π interactions and hydrogen bonds. Specifically, there are four hydrogen atoms on the guest molecule to form multiple CH∙∙∙π interactions with the 1,4-diethoxybenzene subunits of the host. In addition, the two bromine atoms at the periphery of the guest molecule are outside the host cavities and form moderate hydrogen bonds with the hydrogen atoms on the ethoxy groups. It is worth mentioning that we did not find any interaction of the benzoquinone subunit of the host with the guest molecule.

### Host–guest complexation in solution

**Figure 2:**
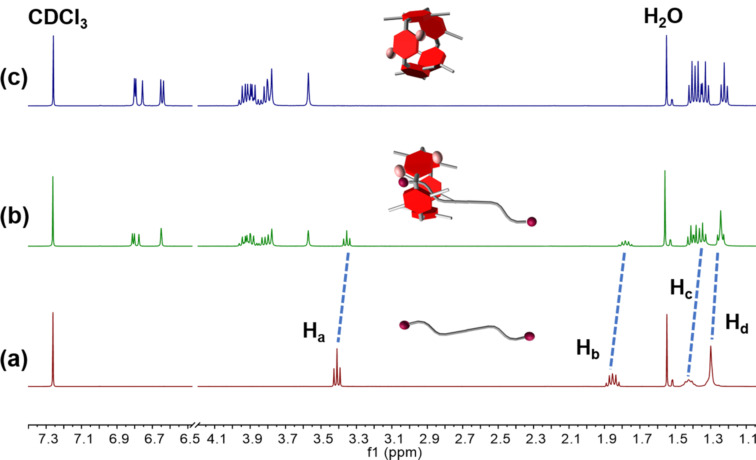
^1^H NMR spectra (500 MHz, CDCl_3_, 298 K): (a) 6.00 mM **G**; (b) 3.00 mM **G** + 3.00 mM **H**; and (c) 6.00 mM **H**.

In order to further study the host–guest binding properties of **H** and **G**, we explored the complexation in solution by ^1^H NMR spectroscopy. As depicted in Figure 2, after the addition of 1.0 equiv of **G** to a solution of **H**, all protons on **G** shifted upfield, which indicated the threading of the alkyl part of the guest into the electron-rich cavity of the host, confirming the complexation between **H** and **G**.

Matrix-assisted laser desorption/ionization time-of-flight mass spectrometry (MALDI–TOF MS) was conducted to investigate the complexation properties. However, no signal related to the complex but only peaks of **H** were found, implying weak host–guest interactions between **H** and **G** (Figures S3 and S4, Supporting Information File 1).

A Job plot based on the proton NMR data was made to determine the complexation stoichiometry between **H** and **G**. The formation signified a 1:1 binding stoichiometry in chloroform-*d* at room temperature (Figures S5 and S6, Supporting Information File 1). Combined with the mass spectrometry results, we believed that the differences between the stoichiometric ratio of the complexation in the solid state and in solution could be owing to the competitive role of solvent molecules in the combination of **H** and **G**. The association constant (*K*_a_) calculated by the nonlinear curve fitting method was 20.0 ± 2.4 M^−1^ (Figures S7 and S8, Supporting Information File 1), which agreed well with the inference above.

**Figure 3:**
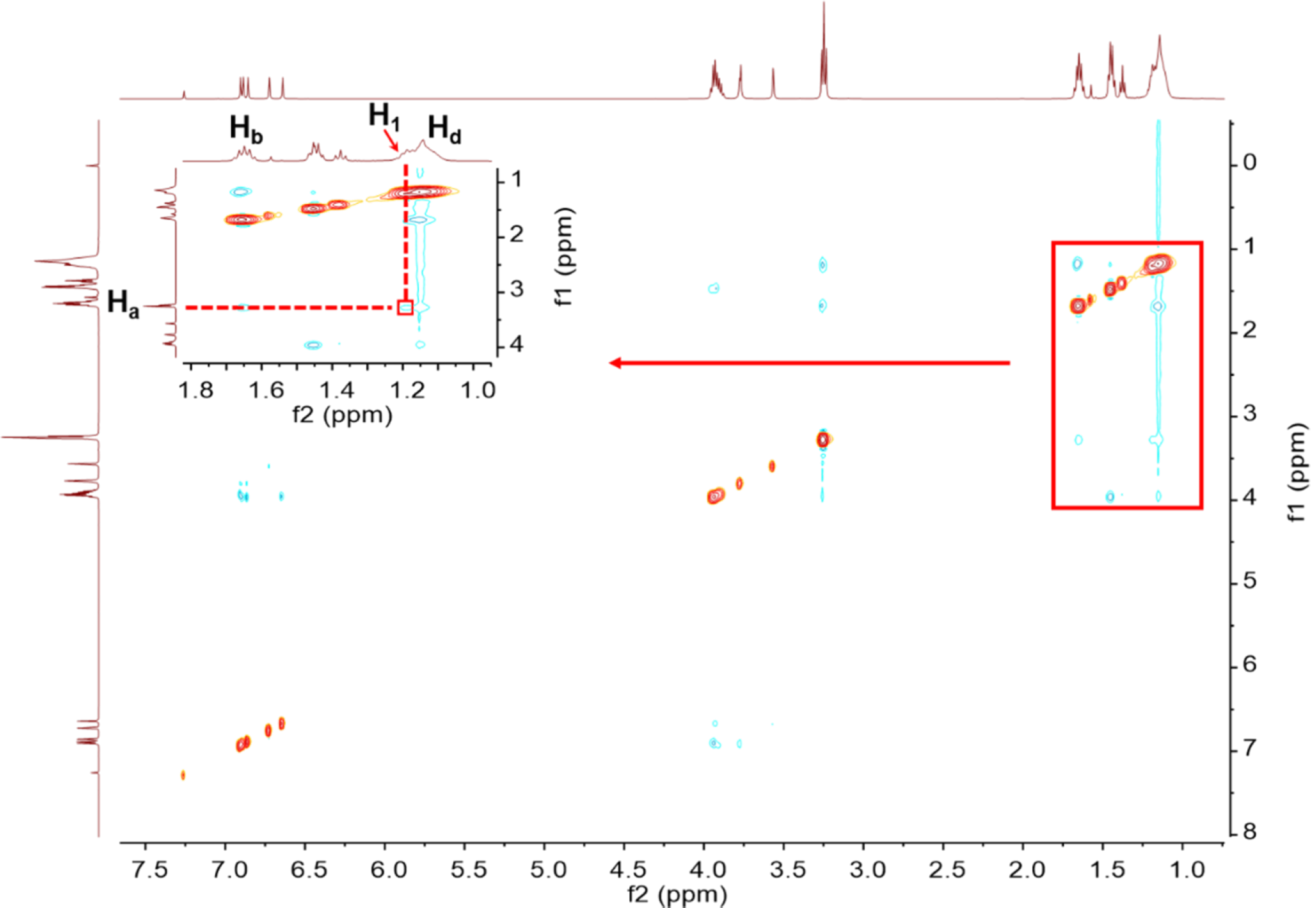
NOESY spectrum of a solution of **H** and **G** (500 MHz, chloroform-*d*, 298 K).

To further explore the geometry of the complexation in solution, we performed a NOESY study (Figure 3). Only one correlative peak was observed between the protons H_1_ of **H** and H_a_ of **G**, which agreed with the formation of the complexation; the alkyl chains of **G** were encapsulated in the cavity of **H**. This further indicated that the interactions between **H** and **G** were weak.

### UV–vis absorption properties of the complex in solution

**Figure 4:**
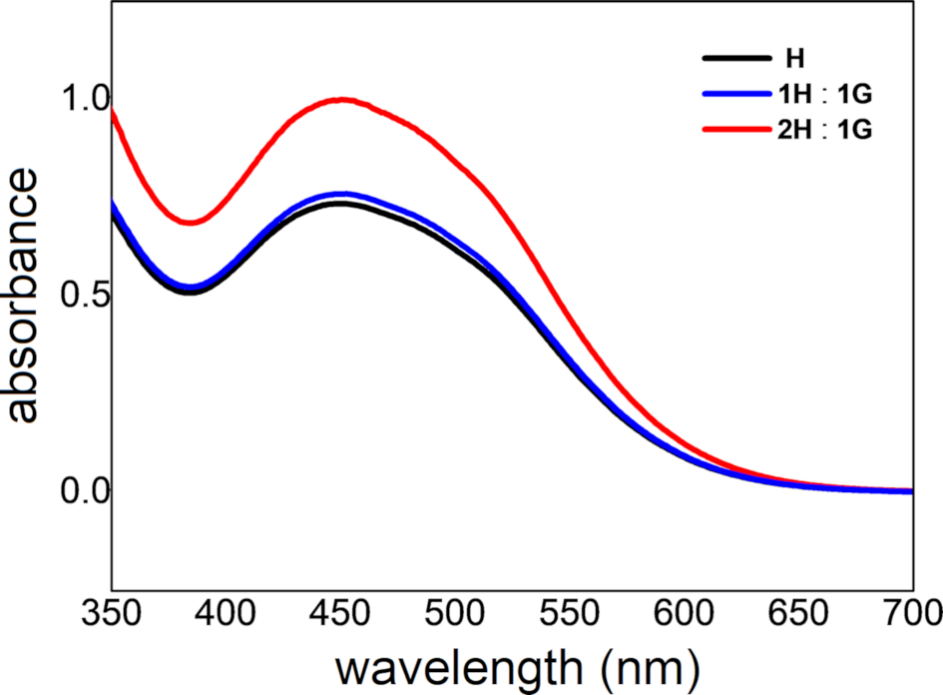
Normalized UV–vis spectra: **H** (black); **H** upon adding 0.5 equiv of **G** (red); and **H** upon adding 1 equiv of **G** (blue). [**H**] = 3.00 mM.

Based on the UV–vis properties of **H**, we wondered the influence of **G** on the optical properties of **H**, so an UV–vis spectroscopy experiment was carried out. We investigated the changes in the UV–vis absorption of the complex at different guest concentrations corresponding to 0.5 and 1 equiv. As shown in Figure 4, the concentration of **G** did not affect the absorption maximum. This also indicated that the interactions between **H** and **G** were weak.

## Conclusion

In summary, we constructed novel pseudorotaxanes based on a pillar[4]arene[1]quinone and 1,10-dibromodecane. Single crystal X-ray diffraction analysis showed an alkane molecule threaded into the cavities of two pillar[4]arene[1]quinone molecules, forming a [3]pseudorotaxane in the solid state. However, ^1^H NMR experiments revealed that the pillar[4]arene[1]quinone encapsulated the guest molecule with a 1:1 stoichiometry to form a [2]pseudorotaxane in solution. One possible reason may be that the interactions between **H** and **G** were weak and that there was a complexation competition with solvent molecules. Furthermore, the addition of **G** did not change the maximum UV–vis absorption wavelength of **H**. The bromine atoms at the periphery of the guest molecule provide convenience for the further capping of the pseudorotaxanes to construct rotaxanes, which will broaden the range of potential applications of pillararene derivatives for the manufacture of sophisticated supramolecular architectures and functional supramolecular systems.

## Supporting Information

File 1 Materials and methods, characterizations of **H** and **G**, crystallographic data, and characterization studies on the complexation between **H** and **G** in solution.

File 2 CIF file for the complex between **H** and **G**.
