# Supplementary material for: Construction of pillar[4]arene[1]quinone–1,10-dibromodecane pseudorotaxanes in solution and in the solid state
Source: Beilstein J Org Chem. 2020 Dec 2;16:2954–9. doi: 10.3762/bjoc.16.245 (PMC7722622; doi:10.3762/bjoc.16.245)
Supplement: File 1 — Materials and methods, characterizations of H and G, crystallographic data, and characterization studies on the complexation between H and G in solution. [file Beilstein_J_Org_Chem-16-2954-s001.pdf]

BEILSTEIN JOURNAL OF ORGANIC CHEMISTRY

Supporting Information

for

Construction of pillar[4]arene[1]quinone–1,10-dibromodecane pseudorotaxanes in solution and in the solid state

Xinru Sheng, Errui Li and Feihe Huang

Beilstein J. Org. Chem. **2020,** *16,* 2954–2959. doi:10.3762/bjoc.16.245

Materials and methods, characterizations of H and G, crystallographic data, and characterization studies on the complexation between H and G in solution

License and Terms: This is a supporting information file under the terms of the Creative Commons Attribution License (https://creativecommons.org/licenses/by/4.0). Please note that the reuse, redistribution and reproduction in particular requires that the author(s) and source are credited and that individual graphics may be subject to special legal provisions.
The license is subject to the *Beilstein Journal of Organic Chemistry* terms and conditions: (https://www.beilstein-journals.org/bjoc/terms)

Table of contents

1. Materials and methods S1
2. Characterizations of **H** and **G** S2
3. Crystallographic data of the [3]pseudorotaxane between **H** and **G** in the solid state S4
4. Characterization studies on the complexation between **H** and **G** in solution S5
5. References S9

1. Materials and methods

All reagents and solvents were commercially available and used as supplied without further purification. **H** was synthesized according to literature procedures.[S1] ^{1}H NMR spectra were recorded on a temperature-controlled 400 MHz, 500 MHz, or 600 MHz spectrometer with use of the deuterated solvent as the lock and the residual solvent or TMS as the internal reference. Matrix-assisted laser desorption/ionization time-of-flight mass spectrometry (MALDI-TOF MS) was recorded on a Bruker Ultraflex spectrometer with a 355 nm Nd: YAG laser (Smartbeam II) and a 25 kV ion source voltage. UV–vis spectra were taken on a Perkin-Elmer Lambda 35 UV-vis spectrophotometer.

2. Characterizations of **H** and **G**

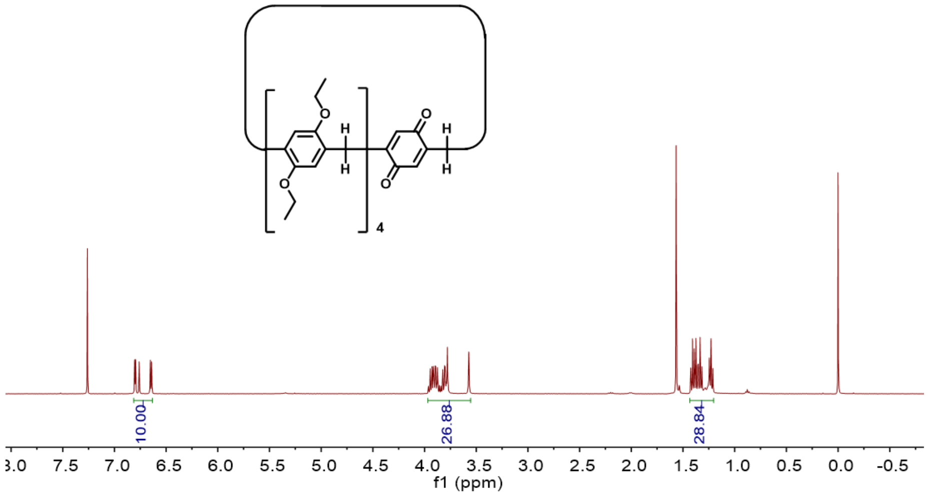

Figure S1: ^{1}H NMR spectrum (400 MHz, $CDCl_3$, 298 K) of **H**.

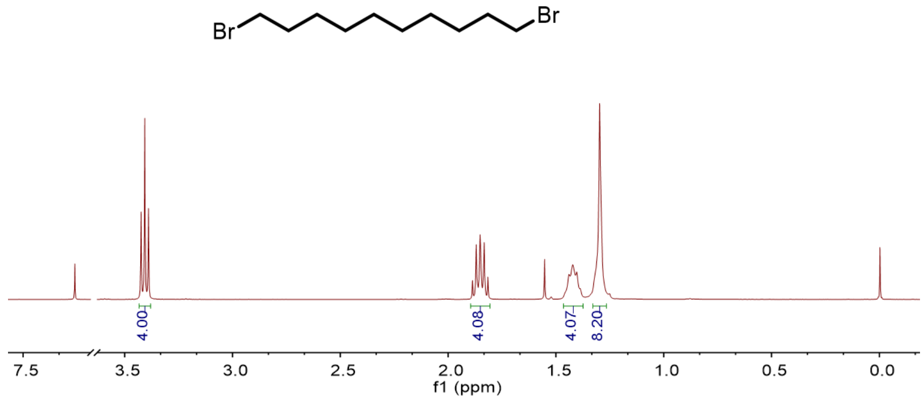

Figure S2: ^{1}H NMR spectrum (400 MHz, $CDCl_3$, 298 K) of **G**.

MALDI-TOF Mass Spectrum

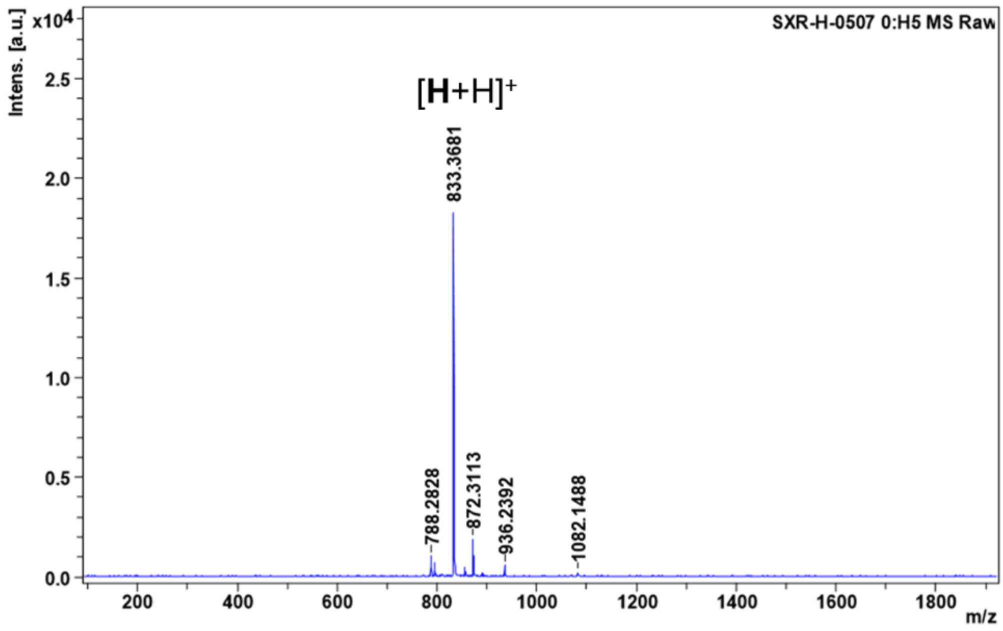

Figure S3: MALDI-TOF mass spectrum of **H**.

3. Crystallographic data of the [3]pseudorotaxane between **H** and **G** in the solid state

Table S1: Crystal data for single crystals of the [3]pseudorotaxane between **H** and **G** in the solid state.

empirical formula	$C_{112}H_{140}Br_2O_{20}$
formula weight	1966.06
temperature	170 K
wavelength	0.71073
crystal system	monoclinic
space group	P 1 $2_1/c$ 1
unit cell dimensions	a = 17.4426(6) Å α = 90<br>b = 19.3528(8) Å β = 96.800(1)<br>c = 15.3931(4) Å γ = 90
volume	5159.6(3)
Z	4
density (calculated)	1.265 g/cm^{-3}
absorption coefficient	0.854
F(000)	2084.0
crystal size	0.336 × 0.139 × 0.116 mm^3
θ range for data collection	2.35 to 27.40°
index ranges	-19<=h<=22, -25<=k<=20, -19<=l<=19
reflections collected	41520
independent reflections	11778 [R(int) = 0.0705]
completeness to θ = 27.492°	0.996
absorption correction	multiscan
max. and min. transmission	0.746, 0.686
data/restraints/parameters	11778/0/612
goodness-of-fit on F2	1.046
final R indices [$I > 2\sigma(I)$]	R1 = 0.0480, wR2 = 0.0511
R indices (all data)	R1 = 0.0705, wR2 = 0.0672
largest diff. peak and hole	0.561, –0.709 e. Å^{-3}
CCDC	2031286

4. Characterization studies on the complexation between **H** and **G** in solution

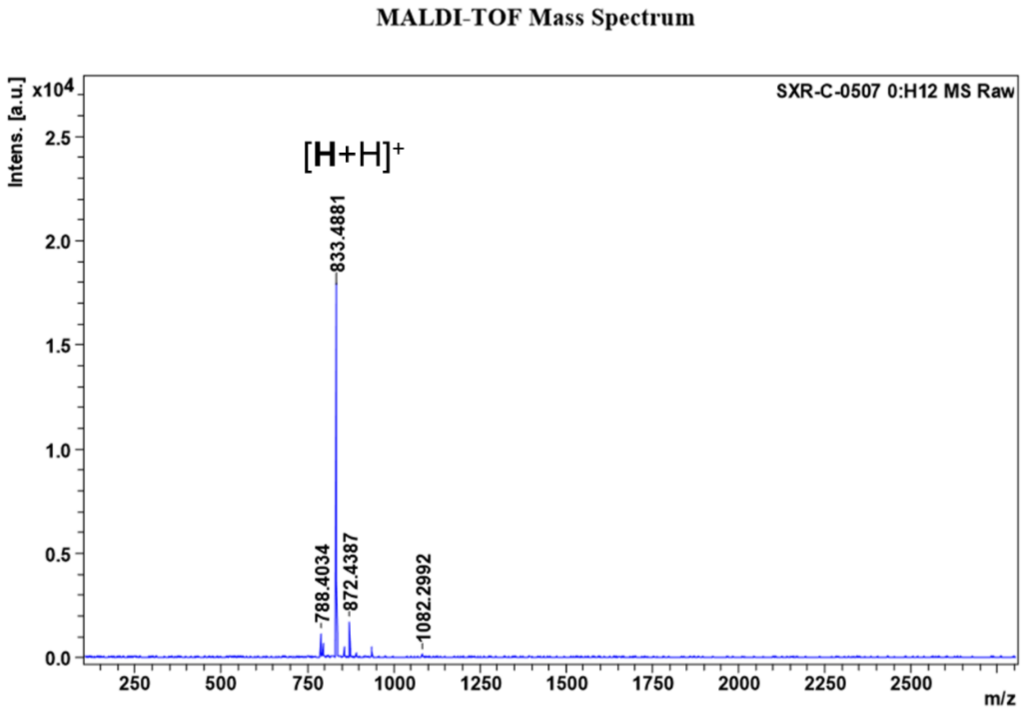

Figure S4: MALDI-TOF mass spectrum of **H** and **G**. There only exist the peaks of **H**.

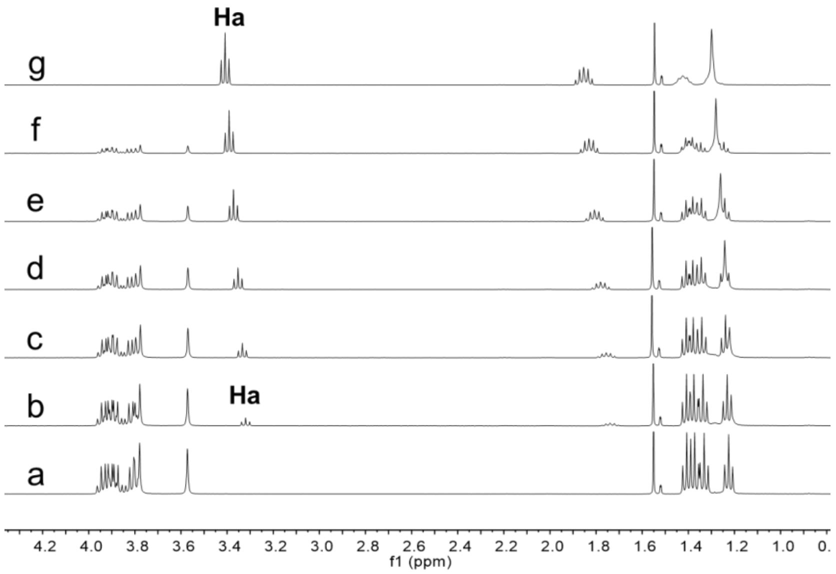

Figure S5: Partial ^{1}H NMR spectra (500 MHz, chloroform-*d*, 298 K): (a) 6.00 mM **H**; (b) 1.00 mM **G** and 5.00 mM **H**; (c) 2.00 mM **G** and 4.00 mM **H**; (d) 3.00 mM **G** and 3.00 mM **H**; (e) 4.00 mM **G** and 2.00 mM **H**; (f) 5.00 mM **G** and 1.00 mM **H**; (g) 6.00 mM **G**.

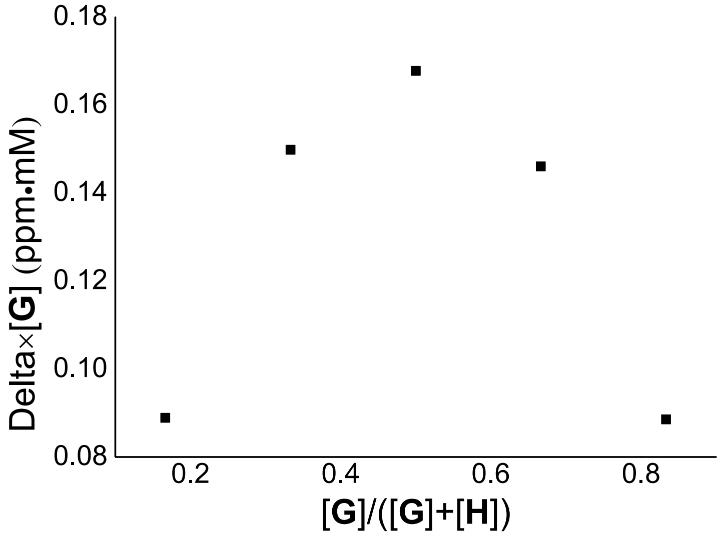

Figure S6: Job plot showing the 1:1 stoichiometry of the complexation between **H** and **G** in chloroform-*d* using the proton NMR data for H_a. Delta is the chemical shift change of H_a. [**H**] + [**G**] = 6.00 mM. [**H**] and [**G**] are concentrations of **H** and **G**, respectively.

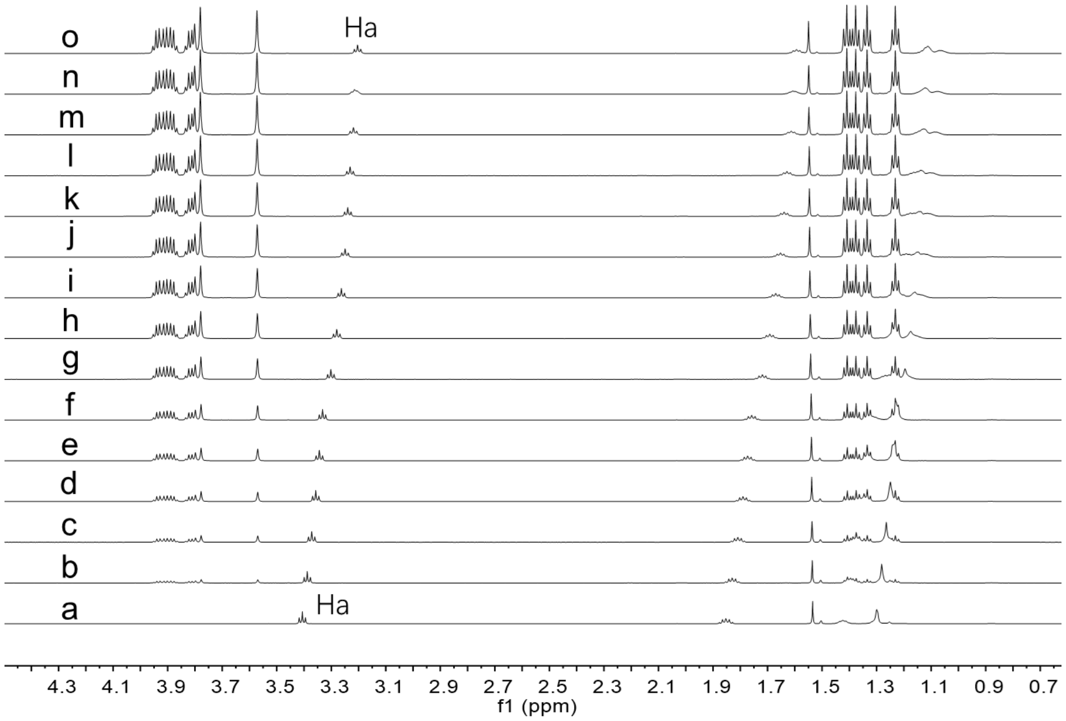

Figure S7: Partial ^{1}H NMR spectra (600 MHz, chloroform-*d*, 298 K) of **G** at the concentration of 5.00 mM upon addition of **H**: (a) 0 mM; (b) 1.16 mM; (c) 2.20 mM; (d) 3.14 mM; (e) 3.98 mM; (f) 4.74 mM; (g) 6.85 mM; (h) 8.47 mM; (i) 9.74 mM; (j) 10.77 mM; (k) 11.63 mM; (l) 12.34 mM; (m) 13.47 mM; (n) 14.33 mM; (o) 15.00 mM.

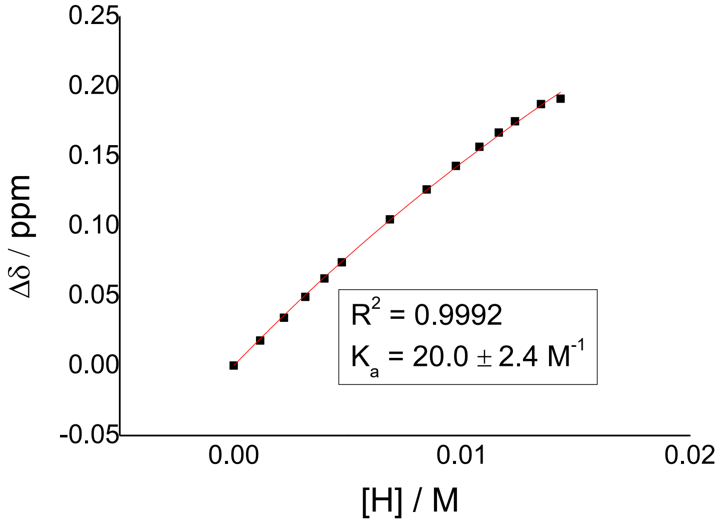

Figure S8: The chemical shift changes upon addition of **H**. The red solid line was obtained from the non-linear curve-fitting equation,[S2] y = (P1/[**G**])(0.5x+0.5 ([**G**]+P2)-(0.5(x^2+(2x(P2-[**G**]))+(P2+[**G**])^2)$^{0.5}$)), where y = Δδ, P1 = $\Delta\delta_{max}$, P2 = 1/K_a, and x = [**H**].

5. References

S1. Han, C.; Zhang, Z.; Yu, G.; Huang, F. *Chem. Commun.*, **2012**, *48*, 9876–9878.

S2. Zhu, X.-Z.; Chen, C.-F. *J. Org. Chem.*, **2005**, *70*, 917–924.
